# Effect of Hyperbaric Oxygen Therapy in Reducing Intestinal Edema After Traumatic Liver Injury with Abdominal Packing: An Experimental Animal Study

**DOI:** 10.3390/life16101675

**Published:** 2026-10-07

**Authors:** Yura Ko, Hye-Eun Byeon, Yo Huh, Jisook Lee, Sehun Choi, Sangchun Choi, Jang-Hee Kim, Heewon Yang

**Affiliations:** 1 Department of Emergency Medicine, Ajou University School of Medicine, 164, World Cup-ro, Yeongtong-gu, Suwon 16499, Republic of Korea; 109427@aumc.ac.kr (Y.K.); eesysook@naver.com (J.L.); simon0978@aumc.ac.kr (S.C.); 2 Institute of Medical Science, Ajou University School of Medicine, Suwon 16499, Republic of Korea; 110236@aumc.ac.kr; 3 Division of Trauma Surgery, Department of Surgery, Ajou University School of Medicine, Suwon 16499, Republic of Korea; ermdhuhyo@aumc.ac.kr; 4 Department of Emergency Medicine, Soonchunhyang University Bucheon Hospital, Soonchunhyang University College of Medicine, Bucheon 14584, Republic of Korea; avenue59@schmc.ac.kr; 5 Department of Pathology, Ajou University School of Medicine, Suwon 16499, Republic of Korea; drjhk@aumc.ac.kr

**Keywords:** hyperbaric oxygen therapy, inflammation, pyroptosis, caspase-1, traumatic liver injury

## Abstract

**Background:** Traumatic liver injury in severe trauma and temporary abdominal closure can lead to postoperative intestinal edema and inflammation. Hyperbaric oxygen therapy (HBOT) may mitigate these effects by modulating inflammatory pathways. This study investigated the effects of HBOT on mucosal preservation, with villus height and the villus-to-crypt (V/C) ratio as primary outcomes and mechanistic analyses of the NLRP3/caspase-1 pathway. **Methods:** Twenty male Sprague–Dawley rats (aged 7 weeks, weighing 260–300 g) were randomly assigned to HBOT (*n* = 10) or non-HBOT groups (*n* = 10) and underwent midline laparotomy (3–5 cm) with 50% left lateral lobe liver resection. After 4 min, the liver was packed with 2 × 2 cm aseptic gauze and the abdomen was temporarily sutured. After 24 h, the small intestine was isolated and flushed with phosphate-buffered saline. The primary outcomes were villus height and the V/C ratio, measured in the proximal jejunum. Secondary outcomes included villus width, crypt depth, villous surface area, and the Dvorak histological injury grade. Additional analyses included NLRP3, AIM2, pro-caspase-1, cleaved caspase-1, IL-1β, and IL-18 expression in bowel tissue (Western blot); plasma IL-1β and IL-18 (ELISA); and peripheral blood mononuclear cell catalase activity, cell viability (CCK-8), and LDH release. **Results:** The HBOT group demonstrated greater villus height compared with the non-HBOT group (347.68 ± 42.48 μm vs. 290.43 ± 43.47 μm; *p* = 0.008), corresponding to a 19.7% increase. The V/C ratio was significantly higher in the HBOT group (2.56 ± 0.31 vs. 2.12 ± 0.23; *p* = 0.002). Villus width was significantly reduced in the HBOT group (64.65 ± 4.24 μm vs. 73.93 ± 9.49 μm; *p* = 0.015). Pathological findings indicated that the HBOT group showed attenuated lamina propria edema and separation. In the intestinal tissue samples, NLRP3 expression was significantly lower in the HBOT group (0.09 [0.09, 0.12] vs. 1.00 [0.92, 1.06]; *p* < 0.001), and AIM2, IL-1β, IL-18, and cleaved caspase-1 followed similar trends. **Conclusions:** HBOT preserved intestinal villus structure after traumatic liver injury, as evidenced by greater villus height and a higher villus-to-crypt ratio. At the molecular level, HBOT suppressed NLRP3, AIM2, and cleaved caspase-1 protein expression in bowel tissue and reduced IL-1β and IL-18 protein levels. By limiting inflammasome activation and interstitial edema, HBOT may help preserve intestinal structural integrity after damage-control surgery.

## 1. Introduction

Severe abdominal trauma is the third most common type of major trauma [1]. Despite advances in trauma resuscitation and surgical techniques, reported mortality after emergency laparotomy for abdominal trauma ranges from 23% to 66.7% depending on the surgical setting [2]. In patients undergoing damage-control surgery for abdominal organ injuries, primary trauma and secondary insults from excessive fluid therapy and systemic inflammation contribute to abdominal organ edema. This can progress to abdominal compartment syndrome and multiple organ failure [3,4,5,6]. As a result, primary abdominal closure is often not feasible, requiring temporary abdominal closure until organ edema resolves. Approximately 25% of patients who undergo abdominal damage-control surgery develop early postoperative small bowel obstruction within 30 days of surgery, resulting in internal organ edema [7]. This leads to prolonged hospitalization, increasing the economic and social burdens.

Hyperbaric oxygen therapy (HBOT) involves the administration of 100% oxygen at pressures greater than the atmospheric pressure, typically in a specialized chamber [8]. This increased pressure enhances oxygen uptake in the lungs, leading to tissue oxygenation. Studies have suggested that HBOT is a promising treatment strategy for anti-inflammation, anti-edema, and amelioration of ischemic reperfusion injury in bowel edema [9,10].

Pyroptosis, which is induced by pathogen- and damage-associated molecular patterns, is associated with increased inflammation mediated by inflammasomes [11]. Reactive oxygen species (ROS), including superoxide anion, hydrogen peroxide, and hydroxyl radical, contribute to oxidative injury and inflammatory signaling. Nuclear Factor kappa B (NF-κB) promotes priming of the NOD-like receptor family pyrin domain-containing 3 (NLRP3) inflammasome, whereas nuclear factor erythroid 2-related factor 2 (Nrf2) regulates antioxidant defenses [12]. In addition to NLRP3, the absent in melanoma 2 (AIM2) inflammasome responds to cytosolic double-stranded DNA [12,13]. Both inflammasomes activate caspase-1, promoting the maturation of interleukin (IL)-1β and IL-18 and the subsequent inflammatory response. HBOT is a well-known treatment strategy for reducing ROS levels [14]. Consequently, the attenuation of NLRP3, caspase-1, and IL-1β results in the attenuation of pyroptosis. HBOT regulates inflammasome NLRP3 activity [11,15]. Pyroptosis induces a rapid decrease in cell viability through cell membrane damage and is accompanied by increased lactate dehydrogenase (LDH) release [11]. Suppression of caspase-1 preserves cell viability, suggesting that pyroptosis plays a direct role in cell death.

Therefore, we hypothesized that HBOT may attenuate intestinal edema and inflammasome-mediated signaling in a rat model of damage-control surgery for traumatic liver injury (50% left-lateral hepatic lobectomy with gauze packing and temporary abdominal closure). The effects of HBOT on intestinal villus structure and the inflammatory signaling pathway were assessed by measuring villus morphometry, NLRP3, cleaved caspase-1, IL-1β, and IL-18 levels in bowel tissue, LDH release, and cell viability.

## 2. Methods

### 2.1. Animals and Study Design

Twenty male Sprague–Dawley rats, aged 7 weeks and weighing 260–300 g, were used in the experiments. The rats were housed under strictly controlled conditions of light and temperature in a specific pathogen-free animal facility at the Laboratory Animal Research Center of Ajou University School of Medicine. They were provided with standard rations and water ad libitum for at least 7 days prior to the experiment. Following the surgical procedure, the rats were fasted until euthanasia. All animal experiments were approved by the Animal Ethics Committee of Ajou University (2023-0001).

The primary outcomes were villus height and the villus-to-crypt (V/C) ratio in the proximal jejunum. Secondary outcomes included additional histomorphometric parameters (villus width, crypt depth, villous surface area, and Dvorak histological grade), intestinal tissue immunoblotting for inflammasome-associated proteins (NLRP3, AIM2, pro- caspase-1, cleaved caspase-1, IL-1β, IL-18), plasma IL-1β and IL-18 concentrations by enzyme-linked immunosorbent assay (ELISA), catalase activity, and cell viability and lactate dehydrogenase (LDH) assay. Bleeding volume at 1, 2, and 4 min after liver injury was recorded to confirm inter-group comparability of the injury model.

The sample size was determined based on the primary outcome of villus height. Assuming a between-group difference of 20% with a standard deviation of 12% based on prior intestinal histomorphometry animal studies, a sample size of 10 rats per group provided 80% power to detect a significant difference at a two-sided α of 0.05 [16,17].

### 2.2. Liver Injury Protocol and Blood Loss Analysis

**Figure 1:**
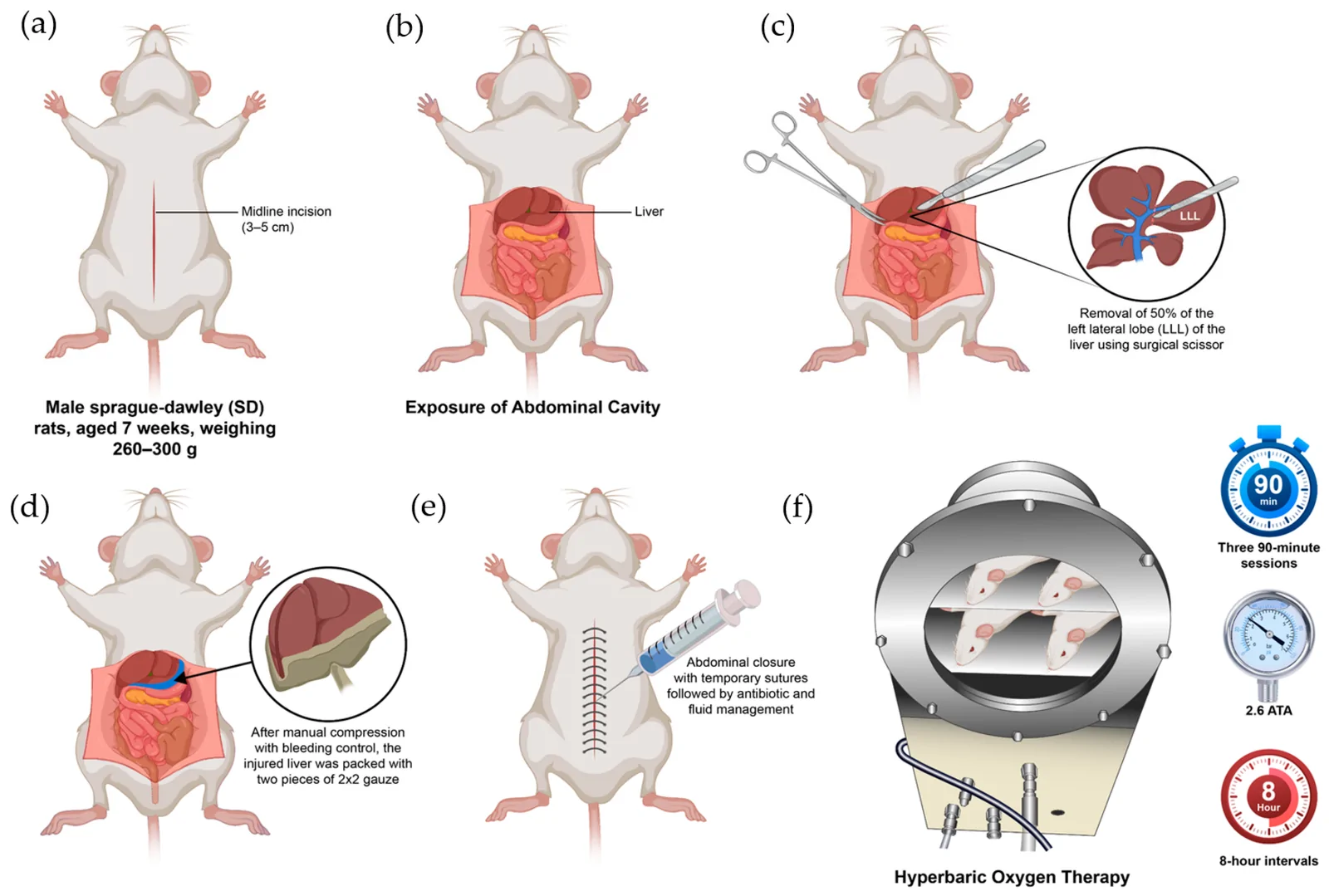
Experimental design for the liver injury and HBOT protocol in rats. (**a**) Male Sprague–Dawley rats, aged 7 weeks and weighing 260–300 g, used in the experiments. (**b**) A midline incision of 3–5 cm made to expose the abdominal cavity. (**c**) Resection of 50% of the left lobe of the liver using surgical scissors. (**d**) Four-minute manual compression with bleeding control, the injured liver was packed with two pieces of 2 × 2 cm gauze. (**e**) The abdominal wall was sutured using an aseptic technique. (**f**) HBOT delivered for 90 min at 2.6 ATA, three times at 8 h intervals over a 24 h period. HBOT, hyperbaric oxygen therapy.

**Figure 2:**
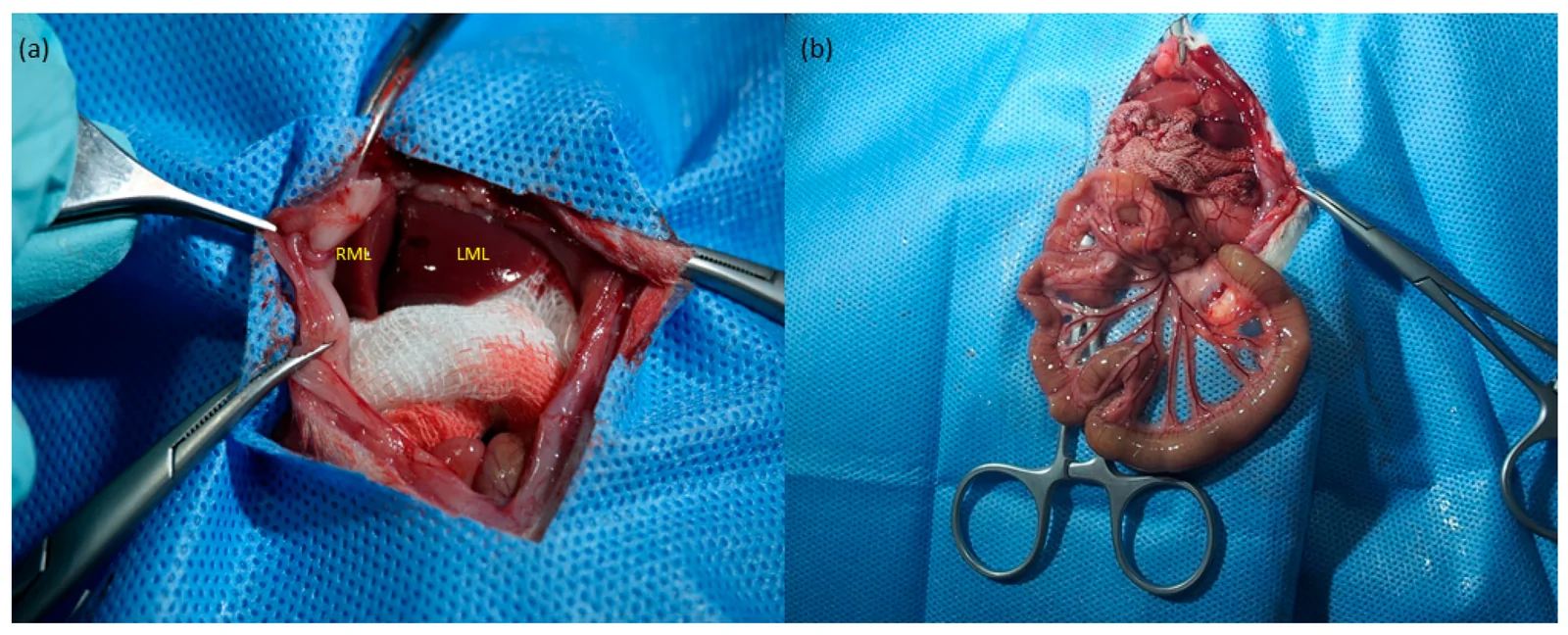
Gauze packing and intestinal ileus after liver injury. (**a**) Fifty percent resection of the left liver lobe, with packing using two 2 × 2 cm sterile gauze pads applied directly to the injury site after 4 min. (**b**) Intestinal ileus after temporary abdominal closure.

A liver laceration model was used in this study. The overall experimental design is illustrated in Figure 1, and representative intraoperative findings are shown in Figure 2. After anesthesia and aseptic preparation of the entire abdominal wall, a midline 3–5 cm incision was made, followed by resection of 50% of the left lobe of the liver using surgical scissors. Four minutes later, the injured liver was packed with two pieces of 2 × 2 cm gauze (Figure 2), and the abdominal wall was sutured temporarily using an aseptic technique [18]. After closing the abdomen, each rat received an intramuscular injection of ceftriaxone (150 mg/kg) and subcutaneous administration of crystalloid solution (0.9% Normal saline, 30 mL/kg) for fluid resuscitation.

Blood loss was quantified during and after the procedure using an aseptic syringe and a 5 mL pipette at 1, 2, and 4 min post-injury, based on a previously validated rat liver laceration model [19]. The total blood volume (TBV) was estimated as 65 mL/kg based on individual body weights. For standardization, blood loss was calculated per body weight (mL/kg) and as a percentage of estimated TBV.

Immediately after blood collection, 18 gauge perfusion needle connected to a peristaltic pump was introduced into the left ventricle, and the right atrium was incised concurrently to establish venous outflow. The systemic vasculature was perfused with ice-cold, heparinized phosphate-buffered saline at a constant flow rate of 5 mL/min until the effluent ran clear and uniform blanching of the liver and small intestine was observed. Complete blanching of the mesenteric and intestinal vasculature was used as the endpoint criterion for adequate clearance of intravascular blood.

### 2.3. Hyperbaric Oxygen Therapy Protocol

The 20 rats were randomly assigned using a computer-based random order generator to either the HBOT or non-HBOT group, with 10 rats per group. HBOT was administered using an HBOT animal chamber (IBEX Medical System, Wonju, Republic of Korea). The first session began immediately after temporary abdominal closure. Following a previously established protocol, HBOT was delivered for 90 min at 2.6 ATA, with three sessions starting at 8 h intervals over a 24 h period. The rats in the HBOT group were placed in the HBOT chamber, where they received 100% oxygen at a compression rate of 5 psi/min [20]. Decompression was performed at the same rate (5 psi/min). The non-HBOT group was exposed to normobaric conditions in a similar chamber on the same schedule to control for hypothermia and experimental stress.

### 2.4. Small Bowel Tissue Sampling

Twenty-four hours after temporary abdominal closure, the rats were euthanized. The entire small intestine was isolated from the duodenojejunal (Treitz) flexure to the ileocecal junction. The intestine was opened along its mesenteric border, mesenteric fat and fecal material were removed, and the bowel was aseptically flushed several times with phosphate-buffered saline. Tissue was sampled from the proximal jejunum, 10 cm distal to the duodenojejunal flexure, and the identical site was used for every rat.

### 2.5. Histomorphometric and Histopathological Analysis

Whole intestinal sections were obtained from the proximal jejunum, 10 cm distal to the duodenojejunal flexure. The samples were fixed in 10% formalin overnight, embedded in paraffin, sectioned, and stained with hematoxylin and eosin.

Intestinal morphological changes were assessed by measuring villus dimensions. In each rat, a single H&E-stained section of the proximal jejunum was scanned at low power to identify well-oriented, longitudinally sectioned villi (both the crypt–villus junction and the villus apex visible in the same plane, with an intact central lacteal). Eligible villi in each section were consecutively numbered, and 10 villi per rat were selected using a computer-generated random-number list, without any pre-selection based on villus size or morphology. This yielded a total of 200 villi from 20 rats (10 HBO group and 10 non-HBO group). All measurements were performed by an investigator blinded to group assignment. Villi measurements were averaged within each rat, and rat-level means were used for statistical analysis [21]. Villus height was quantified as the length from the villus–crypt junction to the apex of the villus. Villus width was measured at the mid-villus level as the side-to-side distance perpendicular to the villus axis. Crypt depth was measured as the distance from the crypt–villi junction to the submucosa. The V/C ratio was calculated by dividing villous height by crypt depth [16,17]. Villous surface area was calculated as villus height × villus width [22].

Histological injury was graded using the Dvorak scoring system (0–4) [23,24]: 0, no damage; 1, slight separation of the submucosa and/or lamina propria; 2, moderate separation of the submucosa and/or lamina propria, or edema in the submucosal and muscular layers; 3, severe separation with severe edema in the submucosal and muscular layers, or regional villous sloughing; 4, loss of villi with necrosis. Dvorak system was selected in preference to ischemia–reperfusion grading scales such as the Park/Chiu score [25], because it grades submucosal and muscular edema, which was the principal finding in this model.

### 2.6. Immunoblotting

Whole intestinal tissues were lysed in radioimmunoprecipitation assay buffer containing protease inhibitor cocktail (Sigma-Aldrich, St. Louis, MO, USA; Cat: #P8215). The total protein concentration was measured using a DC protein assay kit (Bio-Rad, Hercules, CA, USA; Cat: #5000121). The lysates were separated using sodium dodecyl sulfate–polyacrylamide gel electrophoresis and transferred to polyvinylidene difluoride membranes (Millipore, Bradford, MA, USA). The membranes were analyzed by immunoblotting using the appropriate primary antibodies and horseradish peroxidase-conjugated secondary antibodies. The primary antibodies used were anti-NLRP3 (Adipogen, Incheon, Republic of Korea; Cat: AG-20B-0014), anti-AIM2 (Santa Cruz Biotech, Santa Cruz, CA, USA; Cat: SC-293174), anti-IL-1β (R&D Systems, Minneapolis, MN, USA; Cat: #AF-501-NA), anti-IL-18 (Abcam, Cambridge, MA, USA; Cat: #ab191860), anti-caspase-1 (Santa Cruz; Cat: #SC-514), and anti-β-actin (Santa Cruz Biotech; Cat: #SC-47778). The immunoblots were developed by Odyssey Systems (Li-Cor Biosciences, Lincoln, NE, USA), and band intensities were measured using the ImageJ program (version 1.54g; National Institutes of Health, Bethesda, MD, USA) [26]. Immunoblot densitometry values were normalized to β-actin and then to the mean of the non-HBOT samples within the same blot; normalized values from the two blots were pooled (*n* = 10 per group). Between-group comparisons used the exact Mann–Whitney U test, and *p* values across the six proteins were adjusted for multiple comparisons using the Benjamini–Hochberg false discovery rate procedure. Supplementary analyses were performed using intestinal tissue samples from three independent animals per group (Supplementary Figure S1). Total NF-κB and phosphorylated NF-κB (p-NF-κB) were assessed by Western blotting, with GAPDH used as a loading control. Band intensities were quantified by densitometry, and the p-NF-κB/NF-κB ratio was calculated to assess NF-κB phosphorylation.

### 2.7. Enzyme-Linked Immunosorbent Assay

IL-1β (R&D Systems; Cat: #RLB00-1) and IL-18 (R&D Systems; Cat: #DY521-05) levels in plasma were determined using ELISA according to the manufacturer’s protocols.

### 2.8. LDH Release, Catalase Activity, and Cell Viability Assays

Peripheral blood mononuclear cells were first counted, and approximately 3000 cells per well were seeded in a 96-well cell culture plate for LDH release and cell viability assays. The levels of LDH in culture supernatants were determined using the EZ-LDH Assay kit (DoGENBio, Seoul, Republic of Korea) according to the manufacturer’s instructions. LDH activity was expressed in U/L; catalase activity and cell viability were expressed as a percentage of the control group. To determine catalase activity, samples were homogenized in ice-cold assay buffer and centrifuged at 10,000× *g* for 15 min at 4 °C. The supernatant was analyzed using a Catalase Activity Assay Kit (ab83464; Abcam, Cambridge, UK) per the manufacturer’s instructions. Briefly, the sample was incubated with substrate for 30 min at 25 °C, stopped, and reacted with Developer Mix. The absorbance was measured at 570 nm using a microplate reader. Cell viability was analyzed at the indicated times using a D-Plus CCK cell viability assay kit (Dongin Biotech, Seoul, Republic of Korea), following the manufacturer’s protocol. A volume of 10 μL of the CCK-8 reagent was added to each well. After incubation for 2 h at 37 °C, the absorbance at 450 nm (OD450) was measured for each well using a microplate reader (Bio-Rad). For cell viability analysis, samples within each experimental group were pooled, and viability was normalized to the control group, which was set to 100%. Individual measurements are presented as dots in the graph.

### 2.9. Measurement of Tissue ATP Levels

Supplementary analyses were performed using intestinal tissue samples from three independent animals per group. Tissue adenosine triphosphate (ATP) levels were determined using an ATP Colorimetric Assay Kit (BioVision, Milpitas, CA, USA; Cat. No. K354-100), following the manufacturer’s protocol. Briefly, harvested tissue samples were homogenized in ATP Assay Buffer and centrifuged at 15,000× *g* for 10 min at 4 °C to collect the supernatant. To prevent enzymatic interference, samples were deproteinized using a Spin Column. The prepared samples were then incubated with the ATP Reaction Mix for 30 min at room temperature in the dark. The absorbance was measured at 570 nm using a microplate reader. ATP concentrations were calculated using a standard curve, normalized to the total protein content of each tissue sample, and expressed as a percentage relative to the non-HBOT group.

### 2.10. Statistical Analysis

Data are presented as mean ± standard deviation or median [interquartile range], as indicated. Normality was assessed with the Shapiro–Wilk test and equality of variances were assessed with the F test. Normally distributed variables were compared with a two-tailed unpaired t-test, with Welch correction when variances differed; the remaining variables were compared with the Mann–Whitney U test, using the exact distribution when no ties were present. Immunoblot densitometry values were normalized to β-actin and then to the mean of the non-HBOT samples within the same blot, and the normalized values from the two blots were pooled (*n* = 10 per group). Because multiple related outcomes were tested, *p* values were adjusted using the Benjamini–Hochberg false discovery rate procedure, and they were applied separately to the six immunoblot comparisons (NLRP3, AIM2, IL-1β, IL-18, pro-caspase-1, and cleaved caspase-1) and to the five plasma and cellular assays (plasma IL-1β, plasma IL-18, LDH release, catalase activity, and cell viability); adjusted values are reported as q. Statistical significance was set at a two-sided *p* < 0.05. Investigators performing the histomorphometric and tissue analyses were blinded to group allocation. All analyses were performed and figures were prepared using R version 4.6.0 (R Foundation for Statistical Computing, Vienna, Austria).

## 3. Results

### 3.1. Bleeding Volume Analysis After Liver Injury

The mean body weight of the rats was 277.7 ± 3.20 g in the HBOT group and 280.0 ± 5.12 g in the non-HBOT group (*p* = 0.244). All rats survived the experiment. The bleeding volumes 1, 2, and 4 min after injury were compared between the HBOT and non-HBOT groups. In the HBOT group (*n* = 10), the mean bleeding volumes per body weight were 9.43 ± 0.37 mL/kg, 15.16 ± 0.70 mL/kg, and 15.52 ± 0.54 mL/kg at 1, 2, and 4 min, respectively. In the non-HBOT group (*n* = 10), the corresponding values were 9.22 ± 0.74 mL/kg, 15.11 ± 0.74 mL/kg, and 15.43 ± 0.60 mL/kg, respectively. At 1, 2, and 4 min post-injury, the mean bleeding volumes relative to the TBV were 14.5 ± 0.57%, 23.3 ± 1.08%, and 23.9 ± 0.84% in the HBOT group and 14.2 ± 1.14%, 23.2 ± 1.14%, and 23.7 ± 0.92% in the non-HBOT group, respectively (*p* = 0.432, 0.872, and 0.721, respectively). At the end of the experimental protocol, immediately prior to euthanasia, the final body weights were 282.0 ± 7.66 g in the HBOT group and 278.9 ± 4.33 g in the non-HBOT group (*p* = 0.284).

### 3.2. Histomorphometric Preservation of Intestinal Mucosa by HBOT

**Figure 3:**
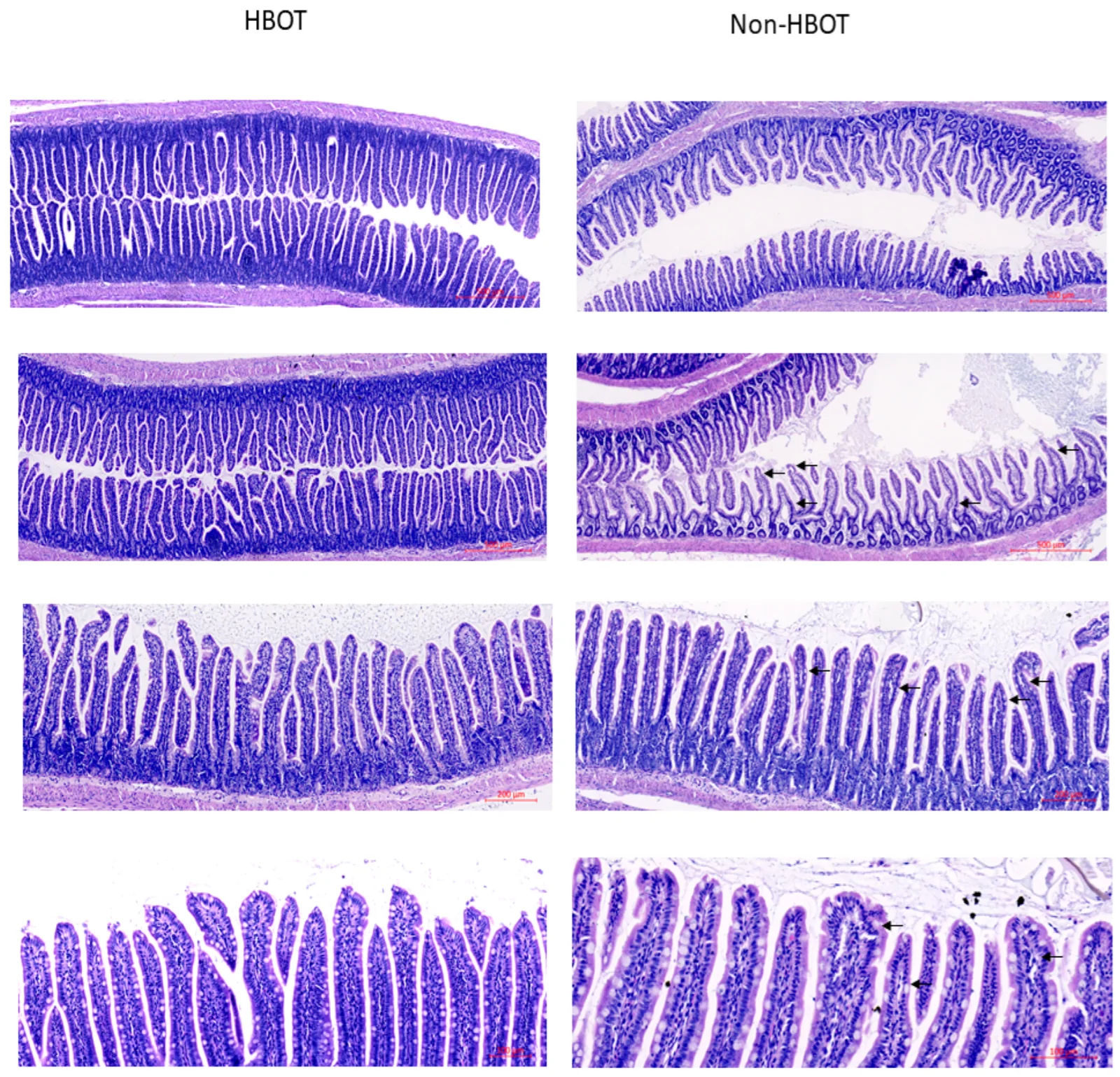
Representative histopathological findings of the small intestinal mucosa in the HBOT and non-HBOT groups. The HBOT group showed reduced diameters of both the outer bowel wall and the inner lumen and lamina propria separation space relative to those in the non-HBOT group. The non-HBOT group showed lamina propria edema and dilation of the lymphatic structures. Arrows indicate lamina propria edema and dilated lymphatic in the non-HBOT group; these findings were attenuated in the HBOT group. HBOT, hyperbaric oxygen therapy.

The histomorphometric parameters of the small intestinal villi are summarized in Table 1. The HBOT group demonstrated greater villus height compared with the non-HBOT group (347.68 ± 42.48 μm vs. 290.43 ± 43.47 μm; *p* = 0.008), corresponding to a 19.7% increase. The V/C ratio, a sensitive indicator of mucosal integrity, was significantly higher in the HBOT group (2.56 ± 0.31 vs. 2.12 ± 0.23; *p* = 0.002). Villus width was reduced in the HBOT group (64.65 ± 4.24 μm vs. 73.93 ± 9.49 μm; *p* = 0.015), consistent with attenuation of lamina propria edema. In contrast, crypt depth (140.39 ± 25.69 μm vs. 139.19 ± 21.96 μm; *p* = 0.912) and total villous surface area (21,698 ± 3719 μm^2^ vs. 21,795 ± 5147 μm^2^; *p* = 0.962) did not differ between the groups. Representative H&E-stained sections are shown in Figure 3. Histologically, the non-HBOT group showed marked edema of the lamina propria and dilation of the lymphatic structures, whereas these changes were attenuated in the HBOT group. The Dvorak histological injury score was significantly lower in the HBOT group than in the non-HBOT group (median 0 [0–0] vs. 2 [2–2]; *p* < 0.001).

### 3.3. Inflammasome-Related Protein Suppression

**Figure 4:**
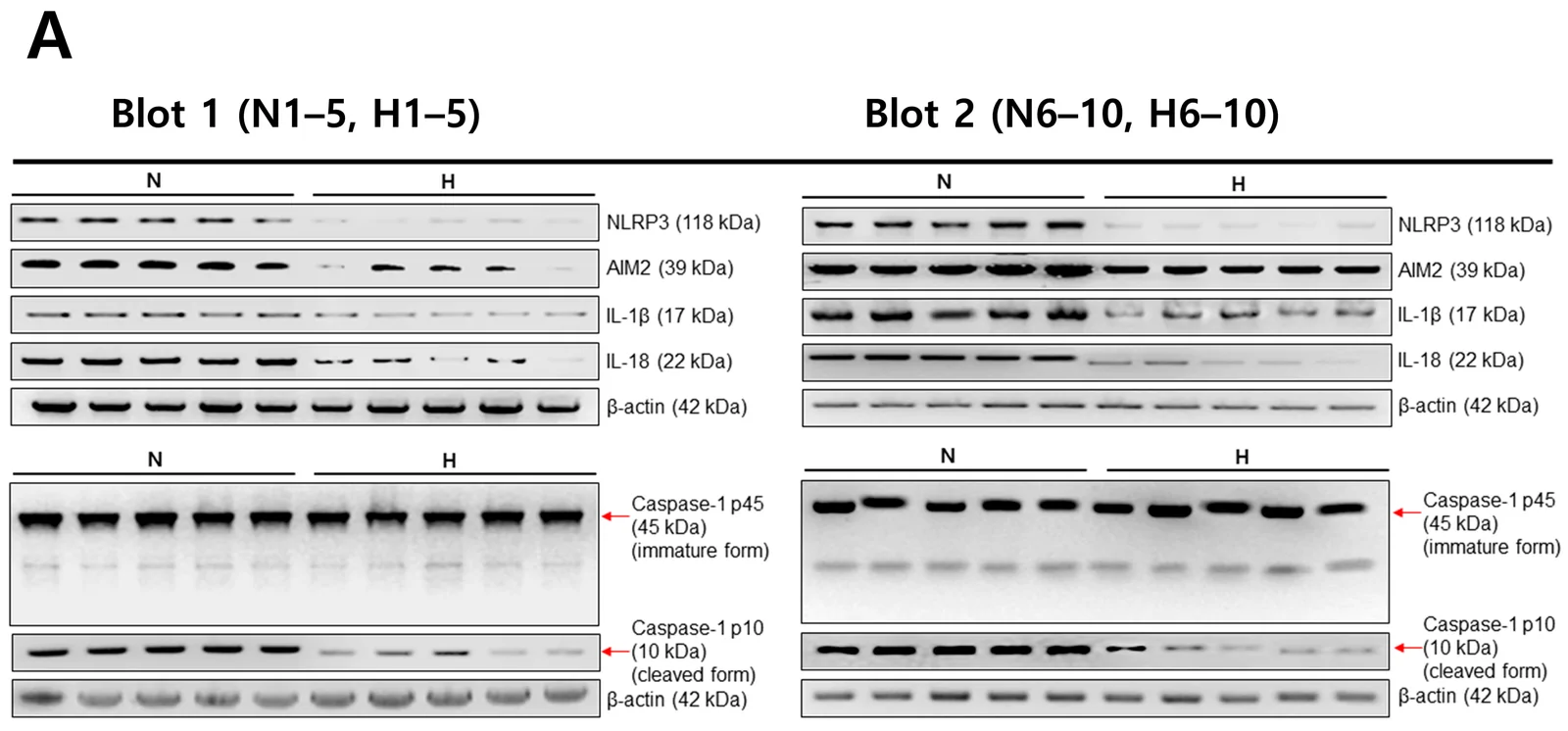

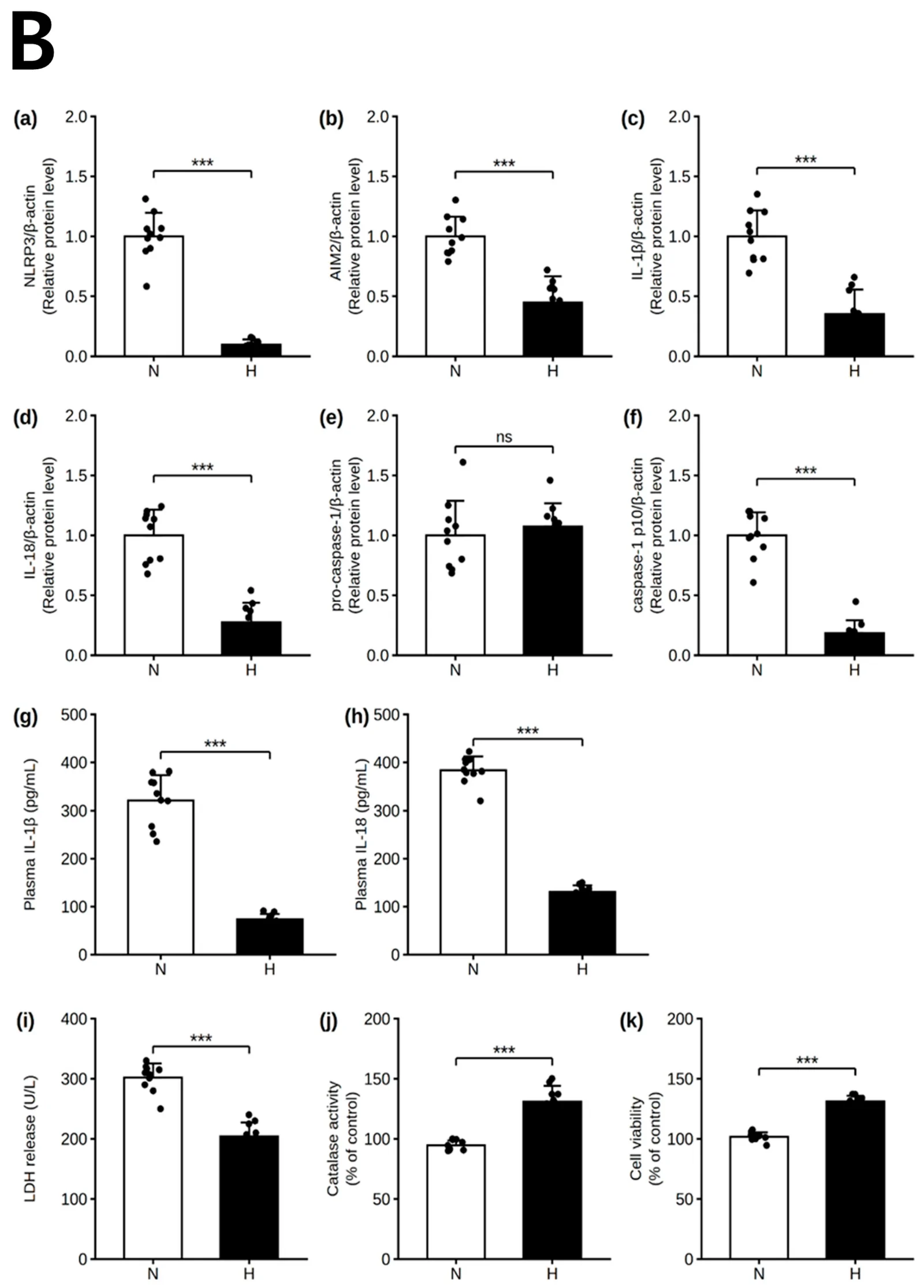
(**A**,**B**) Western blot analysis of intestinal tissue and ELISA measurements of blood samples in the HBOT (H) and non-HBOT (N) groups. (**B**) Western blot analysis of NLRP3 (**a**), AIM2 (**b**), IL-1β (**c**), IL-18 (**d**), pro-caspase-1 (**e**), and cleaved caspase-1 (**f**) in the HBOT (H1–10) and non-HBOT groups (N1–10). Band intensities were normalized to β-actin and then to the mean of the non-HBOT samples within the same blot, and normalized values from the two independent blots were pooled. The Western blots indicate that the HBOT group showed significant suppression of NLRP3, AIM2, IL-1β, IL-18, and cleaved caspase-1 expression compared with the expression in the non-HBOT group, whereas pro-caspase-1 did not differ between the groups. (**B**) ELISA for IL-1β (**g**), IL-18 (**h**), LDH (**i**), catalase activity (**j**), and peripheral blood mononuclear cell viability via CCK-8 (**k**) in the HBOT and non-HBOT groups. The mean expression of IL-1β and IL-18 decreased, while catalase activity and cell viability were activated in the HBOT group, compared with the non-HBOT group. (*p* < 0.001) HBOT, hyperbaric oxygen therapy; ELISA, enzyme-linked immunosorbent assay; CCK, Cell Counting Kit; LDH, lactate dehydrogenase; IL, interleukin; NLRP3, NOD-like receptor family pyrin domain-containing 3; AIM2, absent in melanoma 2. *** *p* < 0.001 versus the non-HBOT group; ns, not significant.

Western blot analysis of inflammasome-associated proteins in intestinal tissue is shown in Figure 4. Two independent Western blot experiments were performed (Blot 1: N1–5 vs. H1–5; Blot 2: N6–10 vs. H6–10). Band intensities were normalized to β-actin and then to the mean of the non-HBOT samples within the same blot, and the normalized values from both blots were analyzed together (*n* = 10 per group). Compared with the non-HBOT group, the HBOT group showed markedly lower expression of NLRP3 (0.09 [0.09, 0.12] vs. 1.00 [0.92, 1.06]), AIM2 (0.52 [0.36, 0.58] vs. 0.97 [0.87, 1.12]), IL-1β (0.35 [0.22, 0.51] vs. 1.00 [0.81, 1.18]), IL-18 (0.28 [0.14, 0.39] vs. 1.10 [0.80, 1.17]), and cleaved caspase-1 (0.15 [0.12, 0.20] vs. 1.00 [0.92, 1.16]); all comparisons remained significant after correction for multiple testing (*p* < 0.001, Benjamini–Hochberg adjusted q < 0.001). In supplementary analyses involving three animals per group, the intestinal p-NF-κB/NF-κB ratio was lower in the HBOT group than in the non-HBOT group (0.133 ± 0.058 vs. 0.967 ± 0.058; *p* < 0.001; Supplementary Figure S1)

### 3.4. Plasma Cytokines, Antioxidant Activity, and Cell Function

Plasma concentrations of IL-1β and IL-18, measured by ELISA, were significantly lower in the HBOT group than in the non-HBOT group (Figure 4B, panels g and h). Plasma IL-1β was 71.35 [66.40–80.25] vs. 328.90 [280.55–358.95] pg/mL, and plasma IL-18 was 130.75 [124.02–137.20] vs. 383.40 [377.85–404.10] pg/mL (*p* < 0.001). LDH release, an indicator of cellular damage, was also significantly lower in the HBOT group than in the non-HBOT group (204 ± 23 vs. 302 ± 23 U/L; *p* < 0.001; Figure 4B, panel i). HBOT significantly increased catalase activity (131.0 ± 13.2% vs. 94.6 ± 4.1%; *p* < 0.001; Figure 4B, panel j). Cell viability was significantly higher in the HBOT group than in the non-HBOT group (131.1 ± 4.8% vs. 101.8 ± 3.6%; *p* < 0.001; Figure 4B, panel k). Intestinal ATP levels were higher in the HBOT group (230.67 ± 5.51% vs. 101.63 ± 4.01%; *p* < 0.001; Supplementary Figure S1c)

## 4. Discussion

Damage-control surgery is a crucial technique for bleeding control and patient stabilization [27]. However, it is associated with several complications. First, packing materials can induce foreign body inflammation in the abdominal organs. Second, the packing procedure can compress vessels that supply the abdominal organs, leading to additional ischemic and reperfusion injuries, which contribute to postoperative ileus and intra-abdominal hypertension. Therefore, damage-control surgery is only a temporary intervention to stabilize patients, with several complications to overcome. Herein, we designed a liver injury model to investigate the effects of liver injury on intestinal edema in rats. In this study, HBOT attenuated intestinal mucosal edema, villus shortening, and inflammasome activation in the rat model.

The bleeding volumes at 1, 2, and 4 min after liver injury, as well as the mean bleeding volume relative to the total blood volume (TBV), did not differ between the HBOT and non-HBOT groups. This finding suggests that the two groups experienced comparable initial hemorrhage, so any subsequent differences between the groups were unlikely to be explained by differences in initial blood loss. The severity of hemorrhage in our model was consistent with previous hemorrhagic liver injury models [28]. In earlier studies, most bleeding occurred within the first 2 min and stabilized by 4 min after injury. A previous rat liver injury model reported a mean TBV loss of 19.8% at 4 min, corresponding to class II hemorrhagic shock [19]. These results support the reproducibility and physiological relevance of our liver injury model.

HBOT exerts a direct hemodynamic effect that may explain the preservation of villus height and the reduction in villus width observed in this study. Under hyperbaric conditions, dissolved plasma oxygen increases approximately tenfold, triggering reflex arteriolar vasoconstriction in non-ischemic tissues. Although this response reduces local blood flow, tissue oxygen delivery is maintained because elevated plasma and tissue oxygen tensions sustain a steep diffusion gradient into hypoxic regions [14]. From a Starling-equation perspective, the resulting decrease in capillary hydrostatic pressure shifts the filtration–reabsorption balance toward reabsorption. Filtration into the interstitium falls while venular and lymphatic outflow are preserved, producing a net efflux of interstitial fluid and an approximately 20% reduction in tissue edema [29]. This mechanism is particularly relevant to the post–damage-control surgery intestine, where ischemia–reperfusion injury increases microvascular permeability and drives lamina propria edema, villus blunting, and the increased villus width observed in the non-HBOT group. Rather than acting through ischemia, HBOT appears to attenuate edema via this hyperoxic-vasoconstriction paradox, supporting the translational relevance of our findings to other ischemia–reperfusion settings [30].

These hemodynamic effects were reflected in the villus structure of the HBOT group. The reduction in villus width likely reflects attenuation of lamina propria edema, consistent with the anti-edematic effect of HBOT [28]. In the non-HBOT group, lamina propria edema was accompanied by dilation of intestinal lymphatic vessels on histology, which likely reflects lymphatic drainage impairment and contributes to further interstitial fluid accumulation. The greater villus height and higher villus-to-crypt ratio in the HBOT group suggest reduced mucosal damage, reflecting the preservation of mucosal structural integrity. Because crypt depth was unchanged, the higher villus-to-crypt ratio reflects the maintained villus height rather than crypt hyperplasia, indicating enhanced structural preservation without evidence of active crypt proliferation in this acute model. Total villus surface area was also similar between groups, reflecting the counterbalancing effects of increased villus height and decreased villus width. Together, these findings indicate that HBOT preserves villus structure primarily by reducing interstitial edema rather than by promoting crypt hyperplasia.

Cellular energy depletion can disrupt ion homeostasis and contribute to tissue swelling, as described in hepatic ischemia and reperfusion [31]. In this study, intestinal ATP content was assessed alongside edema to examine energy status in the affected tissue. This assessment was confined to the intestine, and hepatic energy and metabolic parameters were not evaluated. Tissue ATP content alone does not establish recovery of mitochondrial function. The intestinal ATP findings should therefore be interpreted within the scope of local energy status rather than as evidence of restored hepatic metabolism.

Western blot analysis of intestinal tissue showed significant suppression of NLRP3, AIM2, cleaved caspase-1, IL-1β, and IL-18 in the HBOT group compared with the non-HBOT group. Although the pro-caspase-1 levels showed no significant differences, the caspase-1 did not convert to its active form in the HBOT group, preventing the subsequent cleavage of pro-IL-1β into its mature form, IL-1β. Therefore, we suggest that HBOT attenuates inflammasome activation and downstream markers of pyroptosis following damage-control surgery. The results of the cell viability assay and LDH analysis support these findings. We also found that HBOT attenuated AIM2 expression, which may reflect an inflammatory biomarker potentially involved in the acute phase of the foreign body response [32]. In a mouse model involving intraperitoneal injection of methylmethacrylate beads, AIM2 contributes to the early recruitment of neutrophils, and its deficiency leads to attenuated or postponed neutrophil infiltration, indicating a modified acute inflammatory response to biomaterial implantation.

LDH release and cell viability are commonly used indicators of pyroptosis-induced membrane rupture. During pyroptosis, gasdermin-mediated pore formation leads to LDH leakage into the extracellular space. Elevated LDH levels correlated with inflammation and tissue damage. As pyroptosis progresses, LDH release increases, strongly correlating with a decline in cell viability. In this study, the HBOT group showed increased cell viability and decreased LDH release, suggesting that pyroptosis was inhibited. Pyroptosis leads to a more rapid decrease in cell viability than apoptosis owing to immediate membrane damage. Although apoptosis initially preserves membrane integrity, pyroptotic cells undergo sudden rupture, resulting in the release of inflammatory mediators and leakage of intracellular components [33,34,35]. In this study, suppressed caspase-1 activation and reduced IL-1β and IL-18 levels indicate that the pyroptosis pathway was inhibited in the HBOT group.

The suppression of pyroptosis related pathway may be related to the direct anti-edematic effects of HBOT, along with the simultaneous inhibition of the NLRP3 pathway and ROS. Foreign body reactions also trigger pyroptosis, with monocytes and macrophages playing a central regulatory role. These findings support the step-by-step regulatory effect of the HBOT on the canonical NLRP3 pathway. Danger signals prime NLRP3 and pro-IL-1β expression. A second activation signal drives NLRP3 oligomerization, and caspase-1 activation. Cleaved caspase-1 converts pro-IL-1β to IL-1β and activates IL-18, driving endothelial leakage and the characteristic mucosal edema [36]. HBOT was associated with attenuation of these effects of the canonical inflammasome pathway via changes in NLRP3, caspase-1, IL-1β, and IL-18 expression.

HBOT reduced intestinal NF-κB activation and increased catalase activity, suggesting effects on both inflammatory signaling and antioxidant defenses. Previous study reported that altered mucosal NF-κB activation and reduced intestinal injury after HBOT in an LPS model [37]. Reduced NF-κB signaling may limit NLRP3 priming by decreasing NLRP3 and pro-IL-1β expression [12]. In our study, the lower intestinal p-NF-κB/NF-κB ratio in the HBOT group may indicate reduced NLRP3 priming. ROS also participate in stress signaling through Nrf2, which regulates HO-1, NQO1, and glutathione synthesis [12]. Nrf2 activation can reduce ROS accumulation and NLRP3 priming through NQO1 induction [37]. However, Nrf2 deficiency has also been shown to impair NLRP3 and AIM2 activation, indicating that this relationship is not uniformly inhibitory [13]. Nitric oxide supports physiological signaling, but its reaction with superoxide generates peroxynitrite and reduces NO bioavailability [38]. Excessive peroxynitrite promotes oxidation and protein nitration, whereas lower levels can participate in redox signaling [39].

This study has several limitations. First, anatomical variations in the rat liver and the presence of single-lobe injuries may have affected the reproducibility and applicability of the results. Second, pyroptosis was inferred from cleaved caspase-1, IL-1β, IL-18, and LDH release, but gasdermin D was not measured, so pyroptotic pore formation was not directly confirmed; LDH release is a nonspecific marker of membrane injury. Third, intestinal edema was assessed by histomorphometry rather than by direct measures such as the wet-to-dry weight ratio. Fourth, the study did not fully account for the impact of combined intra-abdominal organ injuries, which may have contributed to systemic inflammatory responses and altered outcomes. The model was based on a controlled liver laceration with packing and did not reproduce direct blunt small-bowel contusion, which commonly accompanies clinical blunt hepatic trauma; the findings therefore apply primarily to intestinal edema induced by damage-control surgery and packing rather than to combined hollow-viscus injury. Fifth, our model did not reproduce the full physiological triad of damage control surgery (acidosis, hypothermia, and coagulopathy), which was not measured, or open-abdomen management with temporary abdominal closure and negative-pressure therapy; resuscitation was limited to crystalloid without blood products. Finally, the potential effects of ischemia–reperfusion injury should be considered, as these may have independently affected tissue damage and inflammatory cascades beyond the primary hepatic injury, with potential differences in implicated pathways from those in human patients.

## 5. Conclusions

HBOT preserved intestinal villus structure after traumatic liver injury, as evidenced by greater villus height and a higher villus-to-crypt ratio. At the molecular level, HBOT suppressed NLRP3, AIM2, and cleaved caspase-1 protein expression in bowel tissue and reduced IL-1β and IL-18 protein levels. Together with increased cell viability and decreased LDH release, these findings suggest that HBOT reduced inflammasome-associated signaling. By limiting inflammasome activation and interstitial edema, HBOT may help preserve intestinal structural integrity after damage-control surgery.

## Figures and Tables

**Table 1:** Histomorphometric parameters of the small intestinal mucosa in HBOT and non-HBOT groups.

	HBOT Group (*n* = 10)	Non-HBOT Group (*n* = 10)	*p* Value
Villus height (μm)	347.68 ± 42.48	290.43 ± 43.47	0.008
Villus width (μm)	64.65 ± 4.24	73.93 ± 9.49	0.015
Crypt depth (μm)	140.39 ± 25.69	139.19 ± 21.96	0.912
Villus/crypt ratio	2.56 ± 0.31	2.12 ± 0.23	0.002
Villus surface area (μm^2^)	21,697.62 ± 3719.29	21,794.87 ± 5147.10	0.962

Values are expressed as mean ± standard deviation. Each value represents the mean of 10 villi measured per rat, averaged across 10 rats per group. Histomorphometry measurements were performed by a pathologist blinded to group allocation.

## Data Availability

The datasets generated and analyzed during the current study are not publicly available because they form part of an ongoing research program, but they are available from the corresponding author on reasonable request.

## Supplementary Materials

The following supporting information can be downloaded at: https://www.mdpi.com/article/10.3390/life16101675/s1, Figure S1: Intestinal NF-κB phosphorylation and tissue ATP levels in the non-HBOT and HBOT groups.
