# Supplementary material for: Effect of Hyperbaric Oxygen Therapy in Reducing Intestinal Edema After Traumatic Liver Injury with Abdominal Packing: An Experimental Animal Study
Source: Life (Basel). 2026 Oct 7;16(10):1675. doi: 10.3390/life16101675 (PMC13705933; doi:10.3390/life16101675)
Supplement: Supplementary file 1 [file life-16-01675-s001.zip › life-4528511-supplementary.pdf]

Supplementary Figure S1. Intestinal NF-κB phosphorylation and tissue ATP levels in the non-HBOT and HBOT groups.

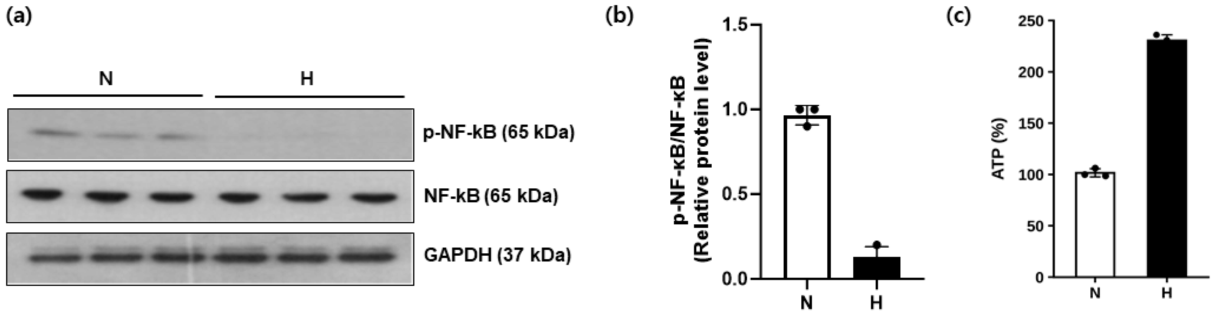

(a) Western blots of phosphorylated NF-κB, total NF-κB, and GAPDH. (b) Densitometric quantification of the p-NF-κB/NF-κB ratio. The p-NF-κB/NF-κB ratio was lower in the HBOT group than in the non-HBOT group (0.133 ± 0.058 vs. 0.967 ± 0.058; $p<0.001$). (c) Intestinal ATP levels were higher in the HBOT group than in the non-HBOT group (230.67 ± 5.51% vs. 101.63 ± 4.01%; $n = 3$ per group; two-sided Welch's t-test, $p < 0.001$).
